# Absence of microRNA-21 does not reduce muscular dystrophy in mouse models of LAMA2-CMD

**DOI:** 10.1371/journal.pone.0181950

**Published:** 2017-08-03

**Authors:** Bernardo Moreira Soares Oliveira, Madeleine Durbeej, Johan Holmberg

**Affiliations:** Muscle Biology Unit, Department of Experimental Medical Science, Lund University, Lund, Sweden; University of Minnesota Medical Center, UNITED STATES

## Abstract

MicroRNAs (miRNAs) are short non-coding RNAs that modulate gene expression post-transcriptionally. Current evidence suggests that miR-21 plays a significant role in the progression of fibrosis in muscle diseases. Laminin-deficient congenital muscular dystrophy (LAMA2-CMD) is a severe form of congenital muscular dystrophy caused by mutations in the gene encoding laminin α2 chain. Mouse models *dy*^*3K*^*/dy*^*3K*^ and *dy*^*2J*^*/dy*^*2J*^, respectively, adequately mirror severe and milder forms of LAMA2-CMD. Both human and mouse LAMA2-CMD muscles are characterized by extensive fibrosis and considering that fibrosis is the final step that destroys muscle during the disease course, anti-fibrotic therapies may be effective strategies for prevention of LAMA2-CMD. We have previously demonstrated a significant up-regulation of the pro-fibrotic miR-21 in *dy*^*3K*^*/dy*^*3K*^ and *dy*^*2J*^*/dy*^*2J*^ skeletal muscle. Hence, the objective of this study was to explore if absence of miR-21 reduces fibrogenesis and improves the phenotype of LAMA2-CMD mice. Thus, we generated *dy*^*3K*^*/dy*^*3K*^ and *dy*^*2J*^*/dy*^*2J*^ mice devoid of miR-21 (*dy*^*3K*^*/*miR-21 and *dy*^*2J*^*/*miR-21 mice, respectively). However, the muscular dystrophy phenotype of *dy*^*3K*^/miR-21 and *dy*^*2J*^/miR-21 double knock-out mice was not improved compared to *dy*^*3K*^*/dy*^*3K*^ or *dy*^*2J*^*/dy*^*2J*^ mice, respectively. Mice displayed the same body weight, dystrophic muscles (with fibrosis) and impaired muscle function. These data indicate that miR-21 may not be involved in the development of fibrosis in LAMA2-CMD.

## Introduction

Laminin-deficient congenital muscular dystrophy type 1A (LAMA2-CMD) is a severe form of muscular dystrophy caused by mutations in the *LAMA2* gene encoding the laminin α2 chain that together with laminin β1 and γ1 chains form the heterotrimeric molecule laminin-211. Without this laminin isoform, the muscle cell loses one of its main connections to the extracellular matrix and a series of deleterious events ensue. General symptoms of LAMA2-CMD include muscle wasting and weakness and delayed motor development [1]. Genotype-phenotype studies have established that complete absence of laminin α2 chain leads to a very severe muscular dystrophy (ambulation is typically not achieved) whereas partial deficiency causes a milder limb-girdle-type muscular dystrophy [2; 3] There are several relevant mouse models for LAMA2-CMD recapitulating grave and milder forms of LAMA2-CMD, including the *dy*^*3K*^*/dy*^*3K*^ and *dy*^*2J*^*/dy*^*2J*^ mouse models. The *dy*^*3K*^*/dy*^*3K*^ mouse completely lacks laminin α2 chain and is the most severely affected among all LAMA2-CMD mouse models, with a life span of approximately 3 weeks. The *dy*^*2J*^*/dy*^*2J*^ mouse has a moderate muscular dystrophy with a significantly longer survival, because it still expresses laminin α2 chain, albeit a truncated chain that is unable to polymerize [2; 3]. Several preclinical approaches to combat LAMA2-CMD in mice have been tested [2; 3]. Addressing the primary cause of the disease and trying to restore the connection between the muscle cell and the basement membrane has been the most successful line of attack, but translation into the clinics remains cumbersome [3–9] Thus, many efforts have also focused on secondary aspects to mitigate disease progression [10–14]. One of the main traits of LAMA2-CMD (as well as other types of muscular dystrophy) is the build-up of fibrotic tissue, which gradually replaces muscle [15–17]. Fibrotic tissue is less elastic and contractile than skeletal muscle, which consequently leads to decreased muscle function. Thus, attempts to prevent or reduce excessive fibrogenesis are highly desirable.

MicroRNAs (miRNAs) are short non-coding RNAs that modulate gene expression post-transcriptionally. Their mature form is about 22 nucleotides long and they work by complementary binding mRNA. Subsequently, miRNA-bound mRNA can be degraded (most cases) or translation is blocked, making miRNAs negative regulators of translation. MiRNA-21 (miR-21) has been implicated in fibrosis in different tissues [18–20]. It is a powerful mediator of fibrogenesis in the *mdx* mouse model of Duchenne muscular dystrophy [21; 22], with its suppression (by antagomirs for miR-21) leading to improved muscle phenotype whereas its over-expression (by miR-21 mimics) worsened it [22]. Furthermore, it was demonstrated that the miR-21 fibrogenic pathway involves PAI-1/urokinase-type plasminogen activator balance and TGF-β [21; 22]. MiR-21 expression is also stress-responsive, as evidenced by its elevated levels after exercise [23] or heart failure [19; 24]. Furthermore, it interacts with major players in fibrosis and inflammation such as TGF-β, Akt, MAPK, Toll-like receptors and osteopontin [19; 21; 25; 26]. Finally, we have demonstrated that miR-21 expression is significantly augmented in *dy*^*3K*^*/dy*^*3K*^ (already at 9 days of age) and *dy*^*2J*^*/dy*^*2J*^ muscle [27].

Therefore, the purpose of this study was to investigate if deleting miR-21 genetically in mouse models of LAMA2-CMD reduces fibrogenesis and improves the phenotype. Hence, we generated *dy*^*3K*^*/dy*^*3K*^ and *dy*^*2J*^*/dy*^*2J*^ mice devoid of miR-21 (*dy*^*3K*^*/*miR-21 and *dy*^*2J*^*/*miR-21 mice, respectively) and analyzed the phenotypes.

## Materials and methods

### Animal models

Laminin α2 chain-deficient *dy*^*3K*^*/dy*^*3K*^ mice were previously described [28]. Heterozygous *dy*^*2J*^*/dy*^*2J*^ (B6.WK-*Lama*^*2dy-2J*^*/*J) [29; 30] and miR-21 ko (B6;129S6-*Mir21a*^*tm1Yoli*^/J) [31] mice were purchased from Jackson Laboratory (Bar Harbor, ME) and bred in the Biomedical Center according to institutional animal care guidelines. Heterozygous *dy*^*3K*^*/+* and *dy*^*2J*^*/+* mice (healthy carriers of LAMA2-CMD), respectively, were mated with miR-21 ko mice. The resulting *dy*^*3K*^*/+*:miR-21*/+* males and females were mated to generate double knockout mice (*dy*^*3K*^*/*miR-21), *dy*^*3K*^*/dy*^*3K*^ mice, miR-21 ko and wild-type (WT) mice. Similarly, the resulting *dy*^*2J*^*/+*:miR-21*/+* males and females were mated to generate double knockout mice (*dy*^*2J*^*/*miR-21), *dy*^*2J*^*/dy*^*2J*^ mice, miR-21 ko and WT mice. Ear biopsies were used for genotyping. All experimental procedures involving animals were approved by the Malmö/Lund (Sweden) Ethical Committee for Animal Research (ethical permit number: M152-14) in accordance with the guidelines approved by the Swedish Board of Agriculture.

#### Animal handling and tissue collection

Three-week-old WT, miR-21 ko, *dy*^*3K*^*/dy*^*3K*^, *dy*^*3K*^*/*miR-21 and 6-week-old WT, miR-21 ko, *dy*^*2J*^*/dy*^*2J*^, *dy*^*2J*^*/*miR-21 mice were euthanized by CO_2_. Quadriceps muscles were dissected for immunohistochemistry and embedded in O.C.T compound (Sakura Finetek, Torrance, CA) prior to freezing in liquid nitrogen or embedded in paraffin. Paraffin embedded specimens were sectioned using microtome (5 μm) (Microm H355, Cellab). Additionally, quadriceps muscles were dissected for hydroxyproline assay and frozen directly in liquid nitrogen.

### RNA isolation and quantitative RT-PCR analysis

Total RNA was isolated from quadriceps muscles from 3-week-old WT, miR-21 ko, *dy*^*3K*^*/dy*^*3K*^ and *dy*^*3K*^*/*miR-21 mice and from 6-week-old WT, miR-21 ko, *dy*^*2J*^*/dy*^*2J*^ and *dy*^*2J*^*/*miR-21 mice using miRNeasy Mini Kit (Qiagen, Valencia, CA), including DNA digestion by DNase I following the manufacturer’s specifications. Concentration and purity of RNA samples were assessed using NanoDrop 1000 Spectrophotometer (ThermoFisher Scientific, Waltham, MA). For miRNA expression analysis, 10 ng of muscle RNA was reverse transcribed to cDNA using the TaqMan Advanced miRNA cDNA Synthesis Kit (Applied Biosystems, Waltham, MA). For mRNA expression analysis, 50 ng RNA was reverse transcribed to cDNA using the High Capacity cDNA Reverse Transcription Kit (Applied Biosystems, Waltham, MA) according to manufacturer’s protocol. The amplification was performed in a LightCycler 480 Real-Time PCR System (Roche Diagnostics, Basel Switzerland) using TaqMan Fast Advanced Master Mix. TaqMan probes detecting mouse miR-21, miR-93 (reference miRNA), TGF-β and Rn18s (reference gene) were used (Applied Biosystems, Waltham, MA). Comparative CT method was used for relative quantitation.

### Histology and morphometric analysis

Muscle sections were stained with hematoxylin and eosin [32] or picro sirius red/fast green [27]. Stained cross-sections were scanned using Aperio’s Scanscope CS2 (with Scanscope console v.8.2.0.1263, Aperio, Vista, CA) and representative images were created using Aperio software. Centrally nucleated muscle fibers representing regenerating muscle cells and peripherally nucleated normal muscle cells were counted in quadriceps muscles using ImageJ software version 1.43u (NIH, Bethesda, MD). A whole area of each muscle cross section was considered.

#### Sirius red/fast green quantification

Collagen content was quantified by a colorimetric method as described by Leon and Rojkind (1985) [33]. Briefly, approximately 15 paraffin sections of 15μm were placed in a plastic tube. Paraffin removal was accomplished by immersing the section in the following solutions: 5 min. xylene, 5 min. xylene/ethanol (1:1), 5 min. ethanol, 5 min. ethanol/water (1:1), 5 min. water. The sections were then stained with fast green/sirius red for 30 min. on rotation. The tissue was washed with distilled water until excess dye is removed and the solution is clear. One ml of 0.1 N NaOH was added and the solutions were analyzed for absorbance at 560 nm and 605 nm.

### Hydroxyproline assay

Quadriceps muscles were isolated and frozen in liquid nitrogen. Samples were weighed and incubated overnight in 200 μl 12 M HCl at 95°C. The hydrolyzate (25 μl) was neutralized with 0.6 M NaOH (25 μl) and incubated with 0.056 M chloramine-T reagent (450 μl) at room temperature for 25 min. Erlich’s reagent (500 μl) was added to each sample and incubated for 1 hour at 65°C, followed by cooling on ice. Subsequently, 100 μl (in duplicates) was transferred to a 96-well plate and absorbance was read at 560 nm. Standards from 4-hydroxyproline at concentrations (μg/μl); 0, 0.05, 0.1, 0.15, 0.2, 0.25, 0.4 were treated the same way as the samples. Absorbance (*A*_560_) of standards was plotted against amount of hydroxyproline (μg) and a linear regression was performed to determine slope and intercept. All absorbance values were subtracted with blank (0 μg/ml hydroxyproline). Content of hydroxyproline in samples was calculated by equation:

$$x(\mu g)=\frac{\left(A_{560}-y_{intercept}\right)}{slope}$$

Collagen conversion factor = 13.5 [27]. Values are presented as μg collagen/mg muscle.

### Grip strength

Forelimb grip strength was measured on a grip-strength meter (Columbus Instruments, Columbus, OH). In short, the mouse was held by the base of the tail and allowed to grasp the flat wire mesh of the pull bar with its forepaws. When the mouse got a good grip it was slowly pulled away by its tail until it released the pull bar. Each mouse was allowed to pull the pull bar five times. The two lowest values were rejected and the mean of the three remaining values was counted. Animals were not subjected to any training prior to the experiment.

### Statistical analysis

The analyses were performed in the Ipython environment [34] using the SciPy (v. 0.18.1) [35] and statsmodels packages (v. 0.61). Difference between groups was assessed by one-way analysis of variance. Multiple test correction was done with the Bonferroni method. Significance was set at the 5% level.

## Results

### The phenotype of *dy*^*3K*^*/*miR-21 double knock-out mice is not improved compared to *dy*^*3K*^*/dy*^*3K*^ single knock-outs

**Fig 1:**
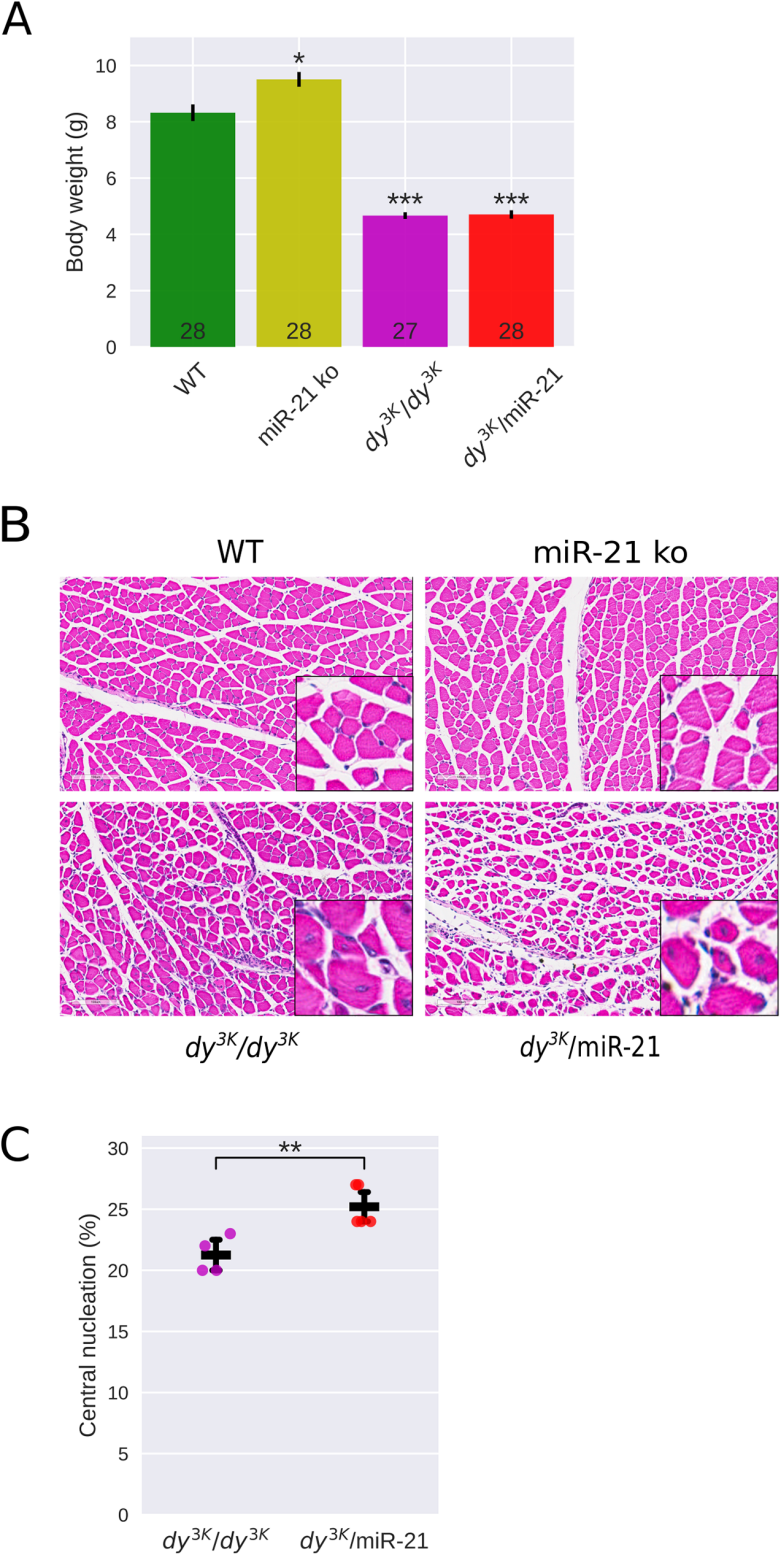
Muscular dystrophy hallmarks are not reduced in 3-week-old *dy*^*3K*^*/*miR-21 mice. **A**. The body weight is significantly increased in miR-21 ko compared to wild-type (WT) mice but significantly reduced in *dy*^*3K*^*/dy*^*3K*^ and *dy*^*3K*^*/*miR-21 mice. Notably, there is no significant difference between *dy*^*3K*^*/dy*^*3K*^and *dy*^*3K*^*/*miR-21 mice. **B**. Hematoxylin & eosin staining of quadriceps muscles shows similar muscular dystrophy histopathology in *dy*^*3K*^*/dy*^*3K*^and *dy*^*3K*^*/*miR-21 muscle. **C**. Quantification of centrally nucleated fibers shows significantly increased number of regenerating fibers in *dy*^*3K*^*/*miR-21 compared to *dy*^*3K*^*/dy*^*3K*^ quadriceps muscle. * p < 0.05, ** p < 0.01, *** p < 0.001.

*Dy*^*3K*^*/dy*^*3K*^ mice display a very severe muscular dystrophy and have a median life span of approximately 3 weeks [3]. In order to investigate if deletion of miR-21 improves the phenotype of *dy*^*3K*^*/dy*^*3K*^ mice, we generated mice (*dy*^*3K*^*/*miR-21) lacking both laminin α2 chain and miR-21 (by series of breeding; please see Materials and methods for further information). Absence of miR-21 in *dy*^*3K*^*/*miR-21 muscle was confirmed by RT-PCR (S1 Fig). The overall health of 3-week-old *dy*^*3K*^*/*miR-21 mice was, however, not improved compared to *dy*^*3K*^*/dy*^*3K*^ mice. *Dy*^*3K*^*/*miR-21 mice exhibited similar decreased survival, growth retardation, muscle wasting, kyphosis and reduced body weight as *dy*^*3K*^*/dy*^*3K*^ mice (Fig 1A).

**Fig 2:**
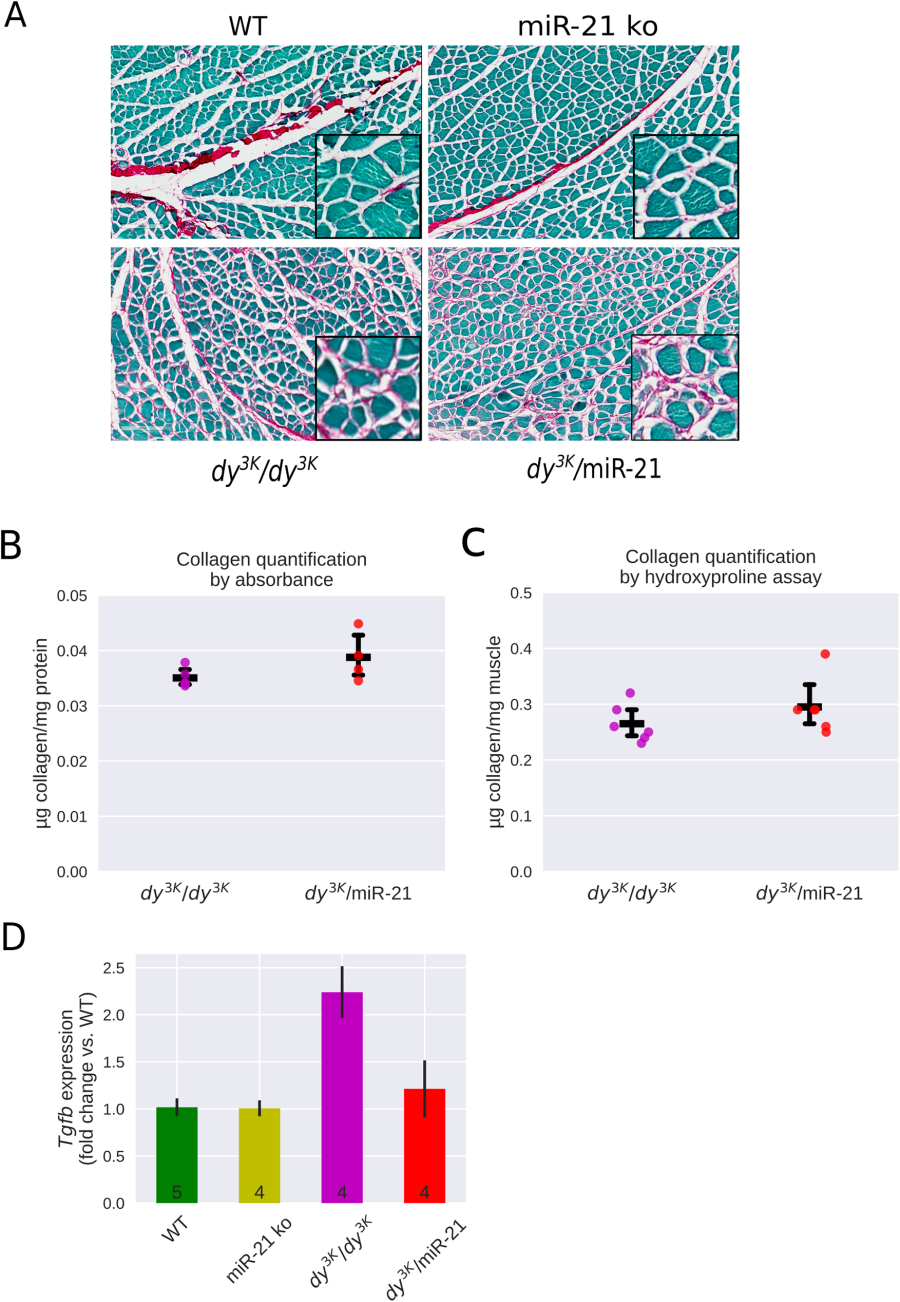
Fibrotic lesions are equally abundant in muscles from *dy*^*3K*^*/dy*^*3K*^ and *dy*^*3K*^*/*miR-21 mice. **A**. Picrosirius red/fast green stained sections demonstrate significant fibrosis (collagen deposition in pink) in *dy*^*3K*^*/dy*^*3K*^ and *dy*^*3K*^*/*miR-21 quadriceps muscle. **B**. Sirius red/fast green quantification of collagen content in *dy*^*3K*^*/dy*^*3K*^ and *dy*^*3K*^*/*miR-21 quadriceps muscle. **C.** Biochemical collagen quantification (hydroxyproline assay) in *dy*^*3K*^*/dy*^*3K*^ and *dy*^*3K*^*/*miR-21 quadriceps muscle. Please note that collagen content is very similar in *dy*^*3K*^*/dy*^*3K*^ and *dy*^*3K*^*/*miR-21 muscle. **D.** qPCR analysis of TGF-β transcript levels in WT, miR-21 ko, *dy*^*3K*^/*dy*^*3K*^ and *dy*^*3K*^/miR-21 quadriceps muscle.

*Dy*^*3K*^*/dy*^*3K*^ muscle is characterized by enhanced apoptosis, degeneration/regeneration cycles, massive inflammation and substantial connective tissue infiltration (fibrosis) [3]. Histological analyses of quadriceps femoris muscle sections from 3-week-old *dy*^*3K*^*/*miR-21 mice revealed that dystrophic alterations were readily present, just like in *dy*^*3K*^*/dy*^*3K*^ muscle (Fig 1B). Regenerating fibers with centrally located nuclei were in fact increased in *dy*^*3K*^*/*miR-21 muscle compared to *dy*^*3K*^*/dy*^*3K*^ muscle (Fig 1C). Measures of fibrosis (sirius red and fast green staining, visualizing collagenous and non-collagenous tissue, respectively, as well as biochemical collagen quantification) demonstrated that a similar deposition of collagen was present in *dy*^*3K*^*/*miR-21 and *dy*^*3K*^*/dy*^*3K*^ muscle (Fig 2A–2C). Finally, expression of TGF-β (a master regulator of fibrosis) has been shown to be enhanced in LAMA2-CMD muscle [16; 36] but analysis of TGF-β gene expression revealed no reduction of TGF-β transcript levels upon miR-21 deletion in *dy*^*3K*^/*dy*^*3K*^ muscle (Fig 2D).

In summary, our results indicated that removal of miR-21 has no beneficial effects in the severely affected *dy*^*3K*^*/dy*^*3K*^ mouse model. Considering that inhibition of miR-21 expression reduced fibrosis in the mildly affected dystrophin-deficient *mdx* mouse model of Duchenne muscular dystrophy [21], we next investigated if removal of miR-21 would have propitious effects in the milder LAMA2-CMD *dy*^*2J*^*/dy*^*2J*^ mouse model.

### The phenotype of *dy*^*2J*^*/*miR-21 double knock-out mice is not improved compared to *dy*^*2J*^*/dy*^*2J*^ single knock-outs

**Fig 3:**
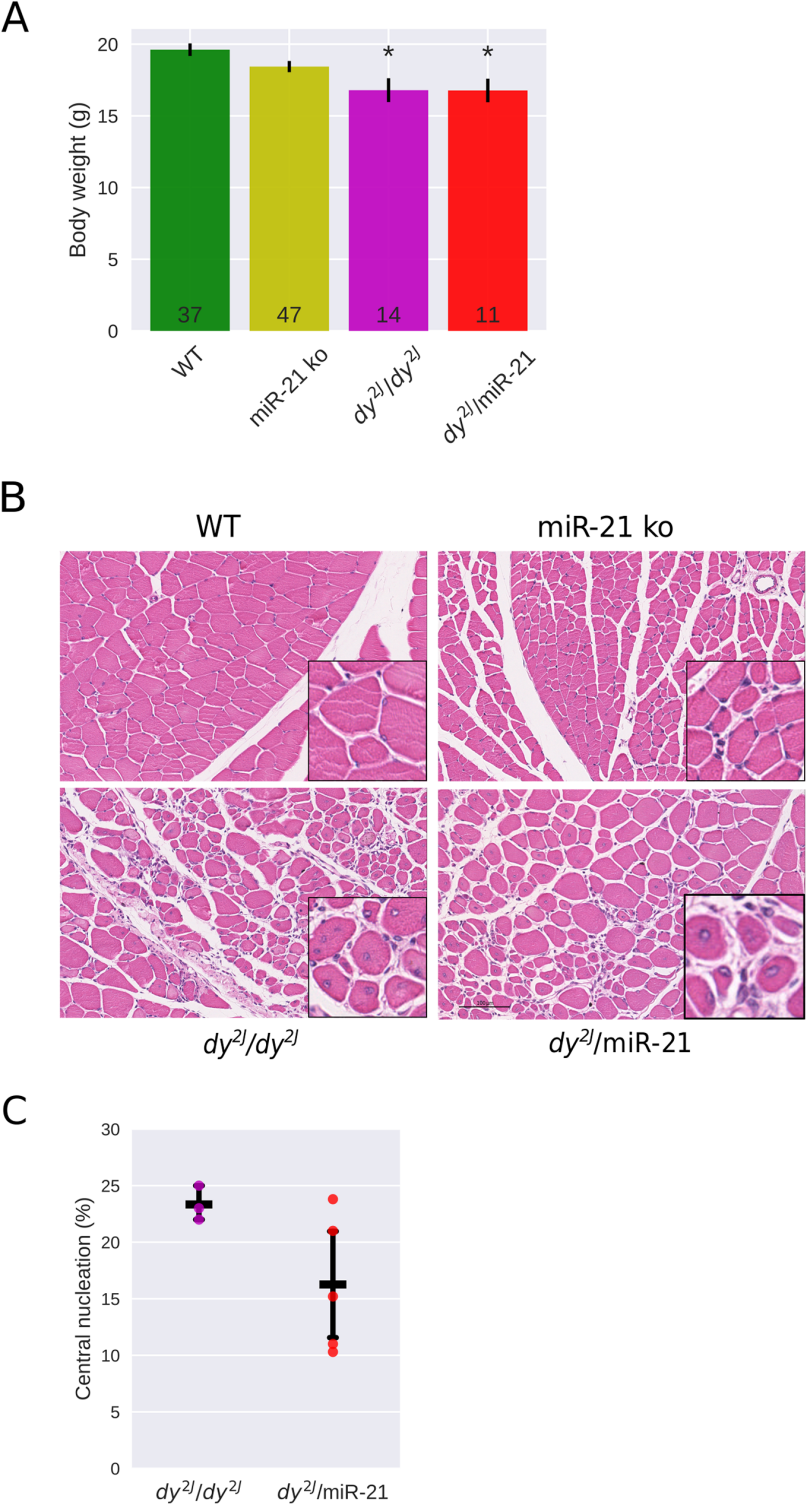
Muscular dystrophy hallmarks are not reduced in 6-week-old *dy*^*2J*^*/*miR-21 mice. **A**. Body weight is significantly reduced in *dy*^*2J*^*/dy*^*2J*^ and *dy*^*2J*^*/*miR-21 compared to WT mice but is not significantly different between *dy*^*2J*^*/dy*^*2J*^ and *dy*^*2J*^*/*miR-21 mice. **B**. Hematoxylin & eosin staining of quadriceps muscles shows similar muscular dystrophy histopathology in *dy*^*2J*^*/dy*^*2J*^ and *dy*^*2J*^*/*miR-21 muscle. **C**. Central nucleation is similar in *dy*^*2J*^*/dy*^*2J*^ and *dy*^*2J*^*/*miR-21 in quadriceps muscle. * p < 0.05.

We generated *dy*^*2J*^*/*miR-21 mice expressing a polymerization-defective truncated laminin α2 chain with no miR-21 (by series of breeding; please see Materials and methods for further information). Similar to the *dy*^*3K*^*/*miR-21 mouse, the overall health of 6-week-old *dy*^*2J*^*/*miR-21 mice was not improved compared to that of *dy*^*2J*^*/dy*^*2J*^ mice and *dy*^*2J*^*/*miR-21 mice exhibited a similar reduction in body weight and hindleg paralysis (Fig 3A).

**Fig 4:**
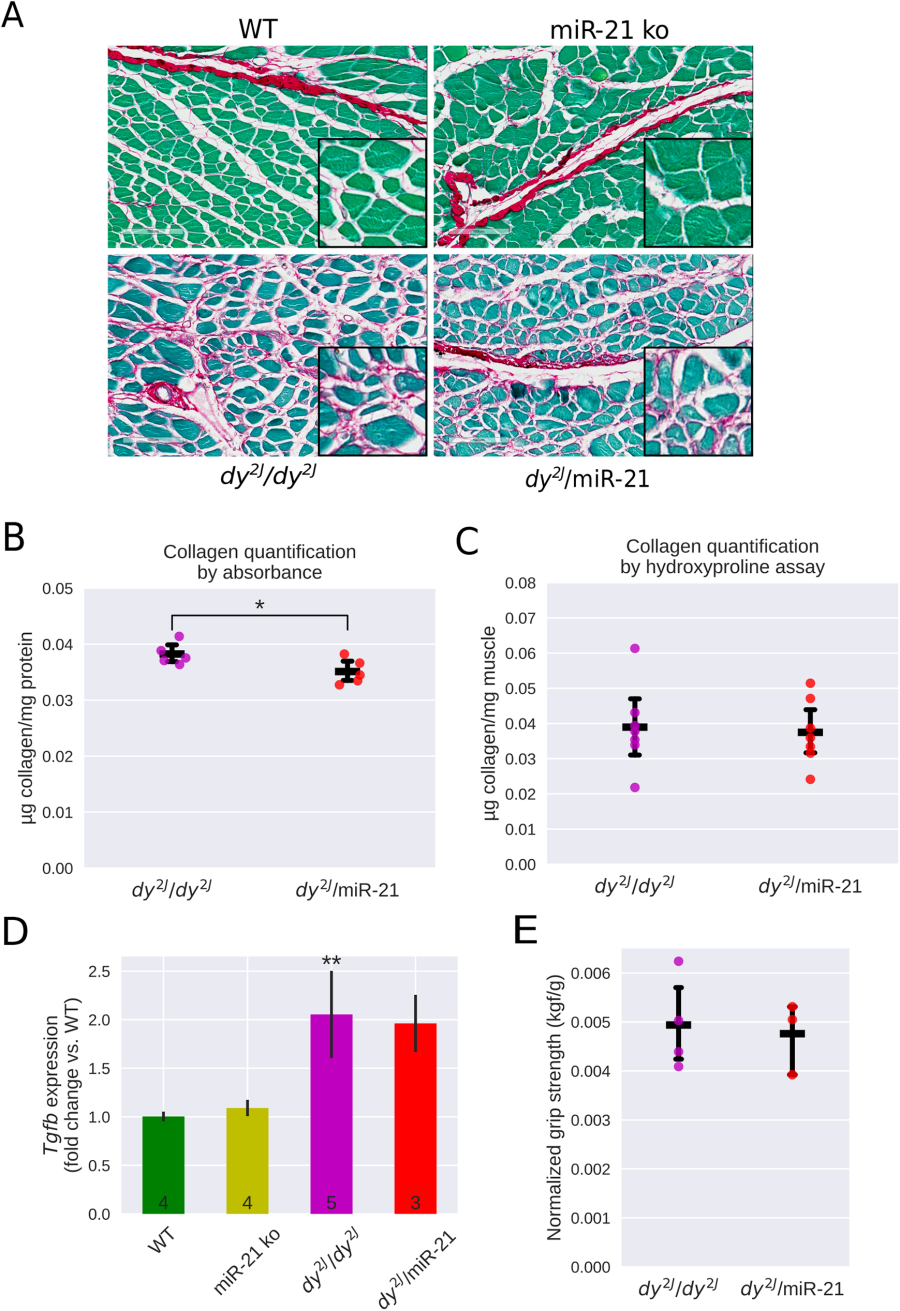
Fibrotic lesions and grip strength in *dy*^*2J*^*/dy*^*2J*^ and *dy*^*2J*^*/*miR-21 mice. **A**. Sirius red/fast green stained sections demonstrate significant fibrosis (collagen deposition in pink) in *dy*^*2J*^*/dy*^*2J*^ and *dy*^*2J*^*/*miR-21 quadriceps muscle. **B**. Sirius red/fast green quantification of collagen content demonstrates slightly reduced fibrosis in *dy*^*2J*^*/*miR-21 compared to *dy*^*2J*^*/dy*^*2J*^ quadriceps muscle. **C**. Hydroxyproline assay reveals no difference in collagen content between *dy*^*2J*^*/dy*^*2J*^ and *dy*^*2J*^*/*miR-21 muscle. **D.** qPCR analysis of TGF-β transcript levels in WT, miR-21 ko, *dy*^*2J*^/*dy*^*2J*^ and *dy*^*2J*^/miR-21 quadriceps muscle. **E**. Grip strength testing uncovers similar forelimb muscle strength (normalized to body weight) in *dy*^*2J*^*/dy*^*2J*^ and *dy*^*2J*^*/*miR-21 mice. * p < 0.05.

*Dy*^*2J*^*/dy*^*2J*^ muscle is also characterized by enhanced apoptosis, degeneration/regeneration cycles, inflammation and connective tissue infiltration [3; 27]. Histological analyses of quadriceps muscle sections from 6-week-old *dy*^*2J*^*/*miR-21 mice revealed that dystrophic alterations were readily present, just like in *dy*^*2J*^*/dy*^*2J*^mice (Fig 3B). Regenerating fibers with centrally located nuclei were present to a similar degree in *dy*^*2J*^*/*miR-21 and *dy*^*2J*^*/dy*^*2J*^ muscle (Fig 3C). Sirius red and fast green staining revealed a similar collagen deposition in *dy*^*2J*^*/*miR-21 and *dy*^*2J*^*/dy*^*2J*^ muscle (Fig 4A), but when quantified, a small but significant reduction in collagen deposition was noted in *dy*^*2J*^*/*miR-21 muscle (Fig 4B). Biochemical collagen quantification (hydroxyproline assay), on the other hand, demonstrated similar collagen accumulation in *dy*^*2J*^*/dy*^*2J*^ and *dy*^*2J*^*/*miR-21 muscle (Fig 4C). Also, expression of TGF-β mRNA was comparable in *dy*^*2J*^/*dy*^*2J*^ and *dy*^*2J*^*/*miR-21 quadriceps muscle (Fig 4D). Finally, we assessed forelimb grip strength. It has previously been demonstrated that grip strength is significantly decreased in 6-week-old *dy*^*2J*^*/dy*^*2J*^ mice, compared to wild-type animals [27]. We found that normalized grip strength (strength divided by body weight) was very similar in *dy*^*2J*^*/*miR-21 and *dy*^*2J*^*/dy*^*2J*^ mice (Fig 4E).

Altogether, our results indicate that removal of miR-21 has no beneficial effects in the severely affected *dy*^*3K*^*/dy*^*3K*^ or in the mildly affected *dy*^*2J*^*/dy*^*2J*^ mouse model of LAMA2-CMD.

## Discussion

The present study aimed at investigating potential benefits of deleting miR-21, a known pro-fibrotic miRNA, in LAMA2-CMD. We present evidence that deleting miR-21 in two different LAMA2-CMD mouse models is insufficient to reduce fibrotic tissue build up and improve the skeletal muscle phenotype. In general, we did not observe any change or improvement due to miR-21 absence, neither on wild-type nor *dy*^*3K*^*/dy*^*3K*^ and *dy*^*2J*^*/dy*^*2J*^ backgrounds, apart from a possible minor reduction in collagen content in *dy*^*2J*^*/*miR-21 muscle. This is in sharp contrast to previous studies on *mdx* mice where inhibition of miR-21 significantly improved disease phenotype [22]. The differences to the aforementioned study could be due to the mouse strains used (*mdx* vs. *dy*^*3K*^*/dy*^*3K*^and *dy*^*2J*^*/dy*^*2J*^) and it is important to note that the *mdx* mouse has mild clinical symptoms in contrast to both *dy*^*3K*^*/dy*^*3K*^ and *dy*^*2J*^*/dy*^*2J*^ mice [2]. Also, the strategy for miR-21 inhibition was different. An antagomir for mir-21 was utilized to silence expression in *mdx* mice whereas a constitutive genetic deletion was used in *dy*^*3K*^*/dy*^*3K*^ and *dy*^*2J*^*/dy*^*2J*^ animals, possibly activating compensatory mechanisms over the course of development. We cannot entirely rule out the possibility that removal of miR-21 could have beneficial effects in older *dy*^*2J*^*/dy*^*2J*^ animals. However, at 6 weeks of age *dy*^*2J*^*/dy*^*2J*^ mice display a full-blown muscular dystrophy but *dy*^*2J*^*/dy*^*2J*^ mice have an extended life span compared to *dy*^*3K*^*dy*^*3K*^ mice (more than 6 months vs. 3 weeks) suggesting that no major worsening of the skeletal muscle phenotype appears after 6 weeks of age.

Despite its bad reputation, inflammation and the ensuing immunological response, is actually fundamental for muscle homeostasis [26]. In healthy tissue there is a fine balance between pro- and anti-inflammatory cues in response to injury and the resolution of the inflammatory response allows for recovery of tissue morphology and function. In LAMA2-CMD, however, this balance is heavily tilted towards pro-inflammatory signals, leading to a state of chronic inflammation [31; 37] Interestingly, miR-21 is also involved in inflammation. Tissue damage leads to increased miR-21 expression through (p38) MAPK [26]. MiR-21 then targets PTEN, an inhibitor of Akt. Relieved from PTEN’s inhibition Akt is free to promote fibroblast proliferation thus increasing collagen synthesis [19–21, 24, 37]. In macrophages the same pathway acts to transition the cells from a pro-inflammatory state into an anti-inflammatory one. In healthy tissue this transition allows the resolution of inflammation and tissue recovery. Under chronic inflammation, however, persistently elevated levels of miR-21 will lead to sustained Akt activity and thus prolonged fibroblast proliferation and delayed transition of macrophages, shortening their anti-inflammatory state [25; 26].

miR-21 has also been shown to promote kidney fibrosis and, in contrast to LAMA2-CMD mouse models, both genetic deletion of miR-21 and inhibition with an antagomir reduced fibrogenesis in mouse models [18]. One explanation to the different outcome in response to miR-21 removal is related to organ-specific aspects of fibrosis. Although various signalling pathways associated with the fibrotic process are conserved across different organs, it is well known that unique, organ-specific features of fibrosis exist [37]. It is also possible that the lack of response upon miR-21 removal in LAMA2-CMD mouse models is due to miRNA promiscuity. That is, one miRNA can target several genes and a single gene can be targeted by many miRNAs. Tarbase (v. 7.0) [38], a manually curated database of experimentally validated miRNA targets, lists 824 unique miR-21a-5p targets, although none in skeletal muscle tissue. MiR-21a-3p has 626 target genes, 160 in common with miR-21a-5p. Moreover, 399 different miRNAs have at least one common target with miR-21a-5p, with 137 having more than 50 common targets. In this scenario it seems very likely that other miRNAs may compensate for miR-21 deficiency. In summary, we have demonstrated that removing miR-21 is not sufficient to improve muscle morphology and function in LAMA2-CMD mouse models. Future research employing multiple miR KOs will have to investigate whether removing other miRNAs that work synergistically with miR-21 represents a better strategy and expanding the list of miR-21 validated targets to include skeletal muscle tissue would help us in that direction.

## Supporting information

S1 Fig miR-21 qPCR. Absence of miR-21 in *dy*^*3K*^/miR-21 mice. Mice with the same miR-21 ko background were used to generate *dy*^*2J*^/miR-21 mice.
(TIF)
