# Supplementary figures and images for: Absence of microRNA-21 does not reduce muscular dystrophy in mouse models of LAMA2-CMD
Source: PLoS One. 2017 Aug 3;12(8):e0181950. doi: 10.1371/journal.pone.0181950 (PMC5542641; doi:10.1371/journal.pone.0181950)

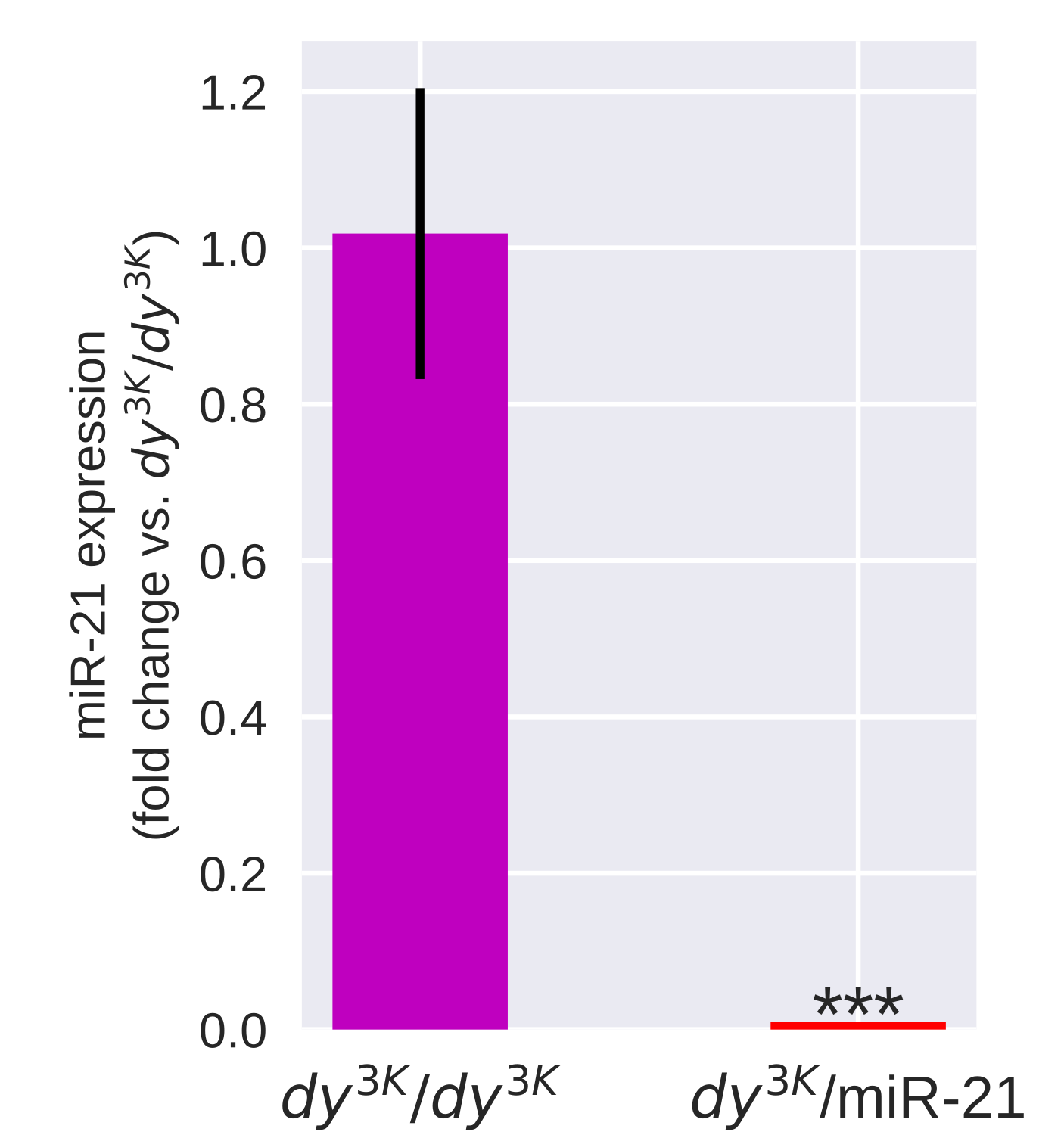

Supplement: S1 Fig — Absence of miR-21 in dy3K/miR-21 mice. Mice with the same miR-21 ko background were used to generate dy2J/miR-21 mice. (TIF) [file pone.0181950.s001.tif]
